# Supplementary material for: Lesion of the hippocampus selectively enhances LEC’s activity during recognition memory based on familiarity
Source: Sci Rep. 2021 Sep 27;11:19085. doi: 10.1038/s41598-021-98509-4 (PMC8476609; doi:10.1038/s41598-021-98509-4)
Supplement: Supplementary file 1 — Supplementary Figures. [file 41598_2021_98509_MOESM1_ESM.pdf]

Supplementary material

Supplementary FIG 1

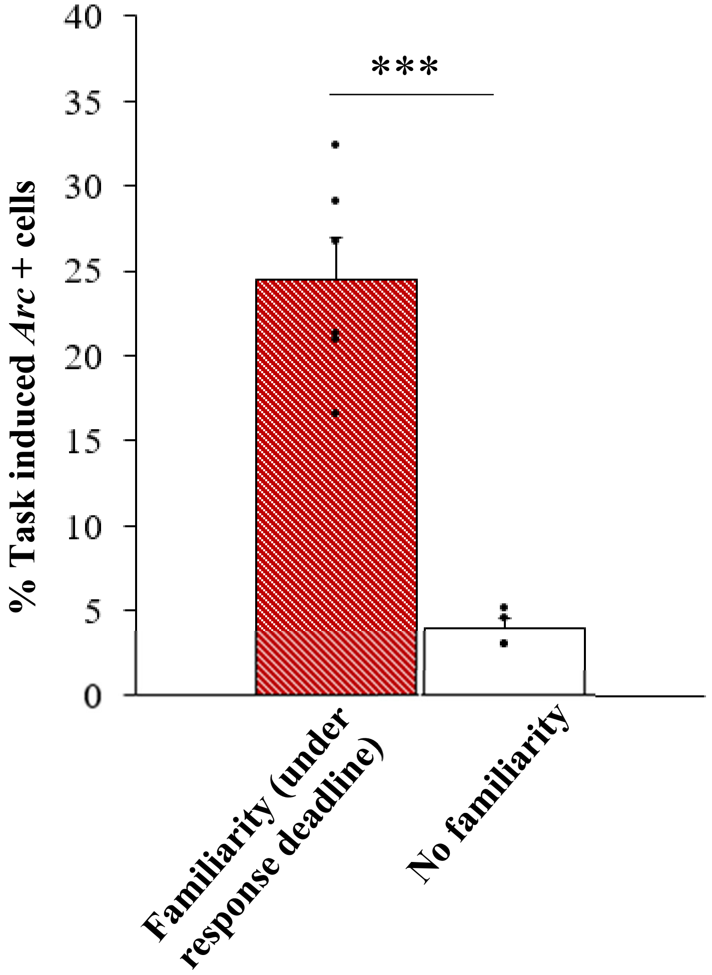

Supplementary Figure 1 (adapted from Atucha et al., 2017: Proportions of *Arc* positive cells in the LEC of rats subjected to a response deadline also leading to familiarity judgments in the DNMS task: evidence that activity in the LEC is tight to the contribution of familiarity to memory performance and not to the contribution of other non-cognitive processes occurring during memory retrieval. Rats were trained to perform the DNMS task described in Fig. 1 but to respond within a response deadline of 2 sec. Implementing this response deadline biases judgments towards relying on familiarity (Sauvage et al., 2010a; Yonelinas and Jacoby 1994; Gronlund et al., 1997; Hintzman et al., 1998; Koen and Yonelinas, 2011) as opposed to relying on both on recollection and familiarity for retrieving memories in animals and in humans. In this study, LEC was overwhelmingly engaged in rats relying on familiarity (light

red bar) when compared to control rats (white bar) that were trained according to the same scheme but were randomly-rewarded instead of following a DNMS rule (i.e. no demands were imposed on the familiarity process for this group). Results indicate that the difference in LEC activity levels between groups likely stems from a difference in the contribution of familiarity to memory performance rather than from the contribution of other non-cognitive processes occurring at retrieval (Familiarity vs No familiarity: $t_{(8)} = 6.747$, $p < 0.001$; comparisons to 0: Familiarity: $t_{(5)} = 16.163$, $p < 0.001$; No familiarity: $t_{(3)} = 7.23$, $p = 0.005$). Of note, both experimental manipulations leading to familiarity judgments in this DNMS task (implementing a response deadline in Atucha et al., 2017 or lesioning the hippocampus in the present study) yield similar levels of activation in the LEC (response deadline vs HIP lesion: $t_{(10)} = 0.724$; $p = 0.486$). Bars represent means ± SEM. *** $p < 0.001$. °$p < 0.001$. Proportions of *Arc* positive cells are normalized by LEC proportions of *Arc* positive cells of respective home-caged control groups.

Supplementary FIG 2

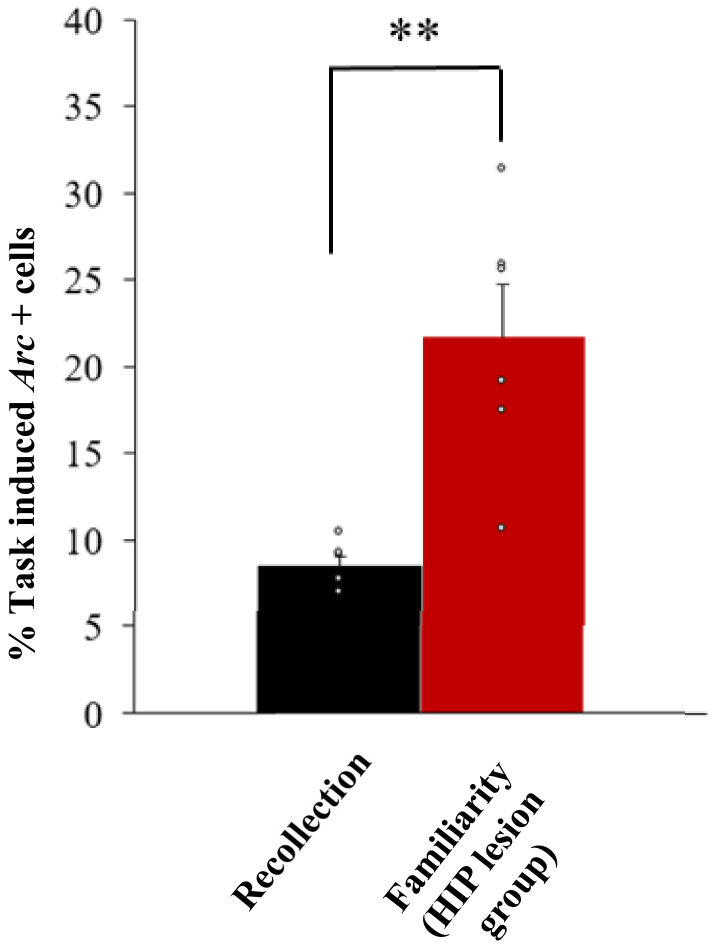

Supplementary Figure 2: Proportions of *Arc* positive cells in the LEC of rats using either recollection or familiarity to solve the DNM task: evidence that activity in the LEC is tight to the contribution of familiarity to recognition memory and not to the contribution of the second cognitive process contributing to recognition memory; recollection. Associative recognition memory, the memory for associated stimuli, relies on recollection in the present DNMS task (Sauvage et al., 2008) whereas performance of rats with hippocampal lesion in the same task relies on familiarity (Fortin et al., 2004). LEC's activity level in rats relying on recollection (black bar) was low whereas it was significantly higher in rats with HIP lesion relying on familiarity (red bar) (Recollection vs familiarity (HIP lesion): $t_{(10)} = 4.29$, $p < 0.01$). This result further supports the claim that activity in the LEC during the retrieval phase of the DNMS task depends on the contribution of familiarity to recognition memory and not on the contribution of other cognitive processes. Bars represent means ± SEM; ** $p < 0.01$;

°$p < 0.001$. Proportions of *Arc* positive cells are normalized by LEC proportions of *Arc* positive cells of respective home-caged control groups.
